# Case Report: Area of focus - involuntary admission for severe Factitious Disorder imposed on self

**DOI:** 10.3389/fpsyt.2025.1649205

**Published:** 2025-09-09

**Authors:** Sim Leng Eng, Hui Ling Michelle Neo, Cheryl Wai Leng Chang

**Affiliations:** Department of Psychological Medicine, National University Hospital, Singapore, Singapore

**Keywords:** Factitious Disorder imposed on self, Munchausen’s syndrome, involuntary admission, civil commitment, case report

## Abstract

Factitious Disorder is clinically challenging to diagnose owing to its reliance on unequivocal evidence of deception. The nature of the disorder and lack of definitive treatment may limit utilisation of mental health legislation, despite its potential fatality. Here, we report a case of a young lady with recurrent, unexplained and life-threatening episodes of iron-deficiency anaemia. Lack of reliable informant to clarify suspicion of Factitious Disorder as well as overshadowing of legal and ethical dilemmas further complicate the clinical management. An involuntary admission, as a last resort, had assisted to solidify the diagnosis of factitious disorder and enforced necessary therapeutic interventions. This Case Report is the first report to highlight the exceptional use of involuntary admission involving a patient with life-threatening factitious anaemia, in Singapore context. It sets the precedence for the exceptional application of the MHCTA outside of gazetted institutions in Singapore, in context of probable severe factitious anaemia.

## Introduction

Factitious Disorder (FD) Imposed on Self is a rare psychiatric disorder with uncertain prevalence in hospital settings, with varying estimated prevalence of 0.02% to 3% across inpatient and outpatient settings (1). The uncertainty in prevalence is partly contributed by its inherent diagnostic challenges (2). Key diagnostic features of FD Imposed on Self, based on the Diagnostic and Statistical Manual of Mental Disorders, Fifth Edition, Text Revision (DSM-5-TR), include falsification of physical or psychological signs or symptoms associated with identified deception, and that the deceptive behaviour is evident even in the absence of obvious external rewards (3). As such, the clinical diagnosis of FD is resource intensive as it would also entail a detailed scrutiny of the medical records and chronology of medical events (2), corroborated by psychosocial investigations to support the possibility of deception and the absence of external rewards.

FD is potentially fatal (4–6) in its most severe form. The exact mortality rate of FD is not known given its rarity and diagnostic challenges. While FD patients often seek hospitalisation, when self-harm behaviours are deemed to be psychologically driven, such as severe hypoglycaemia from self-injection of insulin or severe anemia from bloodletting, they have strong resistance to engage in psychiatric treatment and hospitalisation. This leads to complex clinical and ethical dilemmas regarding the necessity of involuntary admission to safeguard their lives.

In Singapore, three key laws are relevant in the provision of appropriate care and protection to the vulnerable in our society (7):

- The Mental Health (Care & Treatment) Act 2008 (MHCTA)
- The Mental Capacity Act 2008 (MCA)
- The Vulnerable Adults Act 2018 (VAA)

The MHCTA is similar to its older version, the Mental Disorders and Treatment Act 1965, which was modelled on the UK Mental Health Act 1959 (8). It “provides for the admission, detention, care and treatment of mentally disordered persons in designated psychiatric institutions” (9). The term “mental disorder” is broadly defined in Section 2 of MHCTA as “*any mental illness or any other disorder or disability of the mind”* (9), potentially allowing for its utilisation in FD. This Act is helpful for patients who are mentally ill and at significant psychiatric risk but decline voluntary treatment (8). The state mental health hospital, the Institute of Mental Health (IMH), is the only gazetted hospital in Singapore that allows for provision of this act at the point of writing. This leads to a dilemma in cases of patients having both significant medical and psychiatric risk with refusal of treatment (8), as IMH, a tertiary psychiatric hospital, is unable to support patients with complex medical needs. On the other hand, the general hospital medical wards are not designed to treat patients with significant psychiatric risks.

One may then question if the MCA can be utilised in such scenarios. The MCA safeguards vulnerable members of the society who lack mental capacity and allows the enforcement of medical treatment if it is deemed to be in the person’s best interests (10). However, unless considerable work is done to enhance the understanding of both how FD affects capacity and how an FD-induced loss of capacity can be recognised, assessment would be problematic (11). Bass and Halligan (12) argue that deception and pathological compulsion or loss of capacity are mutually exclusive phenomena, suggesting that the loss of control or voluntariness associated with FD cannot accompany the conscious choice associated with common understanding of the phenomena of deception. Further arguments surrounding its ethicality (5, 13, 14) thus complicate provision of involuntary psychiatric care in FD patients on basis of MCA.

In Singapore, the VAA makes provisions for the safeguard of vulnerable adults from abuse, neglect or self-neglect, and provides for matters connected with that (15). The Act defines a vulnerable adult as any individual aged 18 years and above with mental or physical disabilities, and who is unable to protect himself or herself from abuse, neglect, or self-neglect because of these disabilities (7). There are two guiding principles under VAA including (15):

- a vulnerable adult, where not lacking mental capacity, is generally best placed to decide how he or she wishes to live and whether or not to accept any assistance;
- if a vulnerable adult lacks mental capacity, the vulnerable adult’s views (whether past or present), wishes, feelings, values and beliefs, where reasonably ascertainable, must be considered.

The application of VAA thus goes hand in hand with MCA. It is not entirely certain whether it would be helpful in the clinical management of FD patients given its relatively new enactment in 2018 and therefore lack of legal precedence in its application for FD patients in the Singapore context.

To date, there are only a few case reports (16–18) describing the controversial utilisation of civic commitment for FD patients in Western settings. We report a case of probable factitious anaemia, whereby MHCTA was evoked as a last resort to clarify diagnosis and enforce treatment, in Singapore context. It adds more to existing literature and potentially shed light on management of life-threatening FD, where similar medico-legal background may apply.

### Patient information

Ms Y is currently in her mid-20s. She is single, second child in family origin of 4. She is staying with her parents and 2 younger sisters. She has an elder brother, who has been living separately following reports of sexual abuse towards his sisters. Child Protection Services (CPS) assessed that her parents’ poor problem-solving and poor parenting capacity impacted the children’s, especially Ms Y and her brother, emotional regulation and social skills. CPS highlighted concerns of chronic abuse on Ms Y and her sister due to their parents’ ineffective protective strategies and thus the arrangement for her brother to live separately.

Ms Y was able to complete optometry course in technical education, with a low passing score that limits further study. She had worked temporarily in fast food chain, before starting work as a clinic assistant in general practitioner settings. She has been unemployed for the last few years, following the onset of recurrent, symptomatic anaemia necessitating hospitalisation.

**Figure 1:**
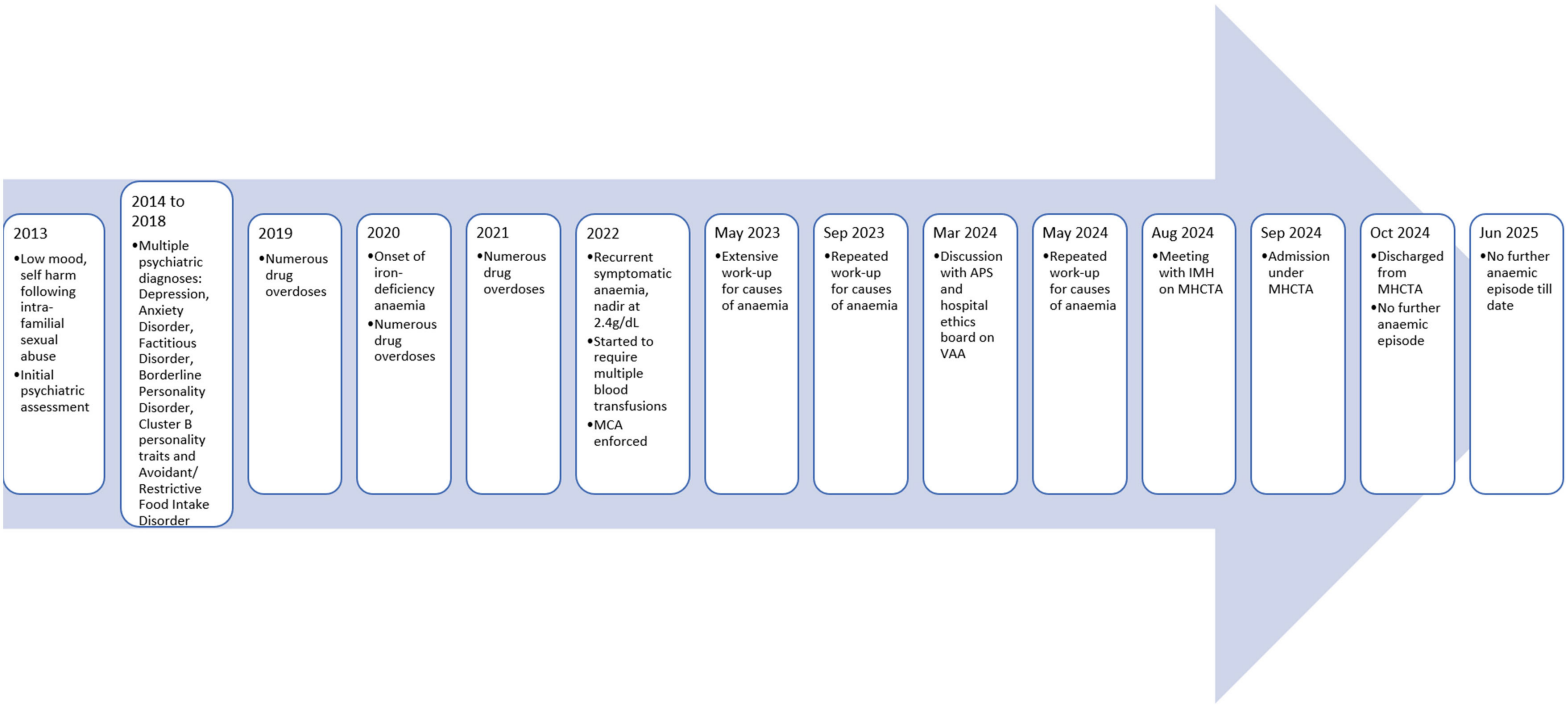
Simplified timeline of clinical presentation and management. *MCA, The Mental Capacity Act 2008; MHCTA, The Mental Health (Care & Treatment) Act 2008; VAA, The Vulnerable Adults Act 2018.

Her psychiatric history began at an early age of 13-years-old. She was brought in by the CPS for psychiatric evaluation as a victim of intrafamilial sexual abuse. She was diagnosed to have Dysthymic Disorder, Hyperventilation Syndrome and Borderline Intellectual Functioning. Her psychiatrist noted poor communication skills and literacy though full IQ test report (done in 2013) is not available at the time of writing. 5 months after initiation of Fluoxetine 10mg/day and psychotherapy sessions, Ms Y was noted to achieve remission. Her conditions fluctuated significantly subsequently, in reaction to stressors such as increased academic demands, school bullying, change in psychiatry multidisciplinary team members, familial discord and interpersonal difficulties. There were recurrent non suicidal self-harm behaviours, escalating in severity from wrist laceration requiring surgical debridement and closure to neck laceration and potentially lethal drug overdoses (refer **Figure 1**). Functional analysis of self-harm behaviour was not feasible due to vague responses and intermittent selective mutism during consultation. Her treating psychiatrist hypothesised these self-harm behaviours stem from need for validation, that Ms Y did not get from home or school.

Multiple psychiatric conditions including Depression, Anxiety Disorder, Factitious Disorder, Borderline Personality Disorder and Cluster B personality traits were added on over the 10-years of follow-up. Comorbidity of Avoidant/Restrictive Food Intake Disorder was added following onset of severe anaemia.

The pharmacological treatments of her mood disorder with various SSRIs, as well as a trial of Quetiapine monotherapy, had been unsuccessful, in part contributed by her reluctance to adhere to medication despite extensive psychoeducation. There was also safety concern in dispensing psychotropics stemming from multiple incidents of drug overdoses. Her psychotherapy progress was frequently disrupted by sudden medical needs and poor attendance, leading to eventual discharge from the psychology service in 2021.

Ms Y’s medical records show 58 hospital admissions, over 150 emergency department attendances, and more than 1500 outpatient visits with various specialties including psychiatry, psychology, general surgery, trauma team, hand & reconstructive microsurgery, haematology, dermatology, gastroenterology, dietetics, occupational therapy, and primary care clinics. She has been diagnosed with the following medical conditions:

- Migraine without aura
- Bilateral breast fibroadenoma
- Anaphylactic reaction to intravenous iron
- Right wrist De Quervain’s tenosynovitis
- Paronychia of left thumb, left middle finger, left toe in different years
- Recurrent right ankle sprain requiring arthroscopic debridement and arthrobrostrom and peroneal tendinoscopic debridement in 2022
- Left ankle anterior talofibular ligament sprain requiring arthroscopic debridement and arthrobrostrom in 2023
- Multiple drug overdoses of non-lethal intent across 2019 to 2021
- Deliberate self-harm via slashing wrist, neck, chest, flank across 2020 to 2023

Notably, Ms Y was diagnosed with FD imposed on self in 2020 after she confessed to removing stitches from her abdominal wound on multiple occasions.

**Figure 2:**
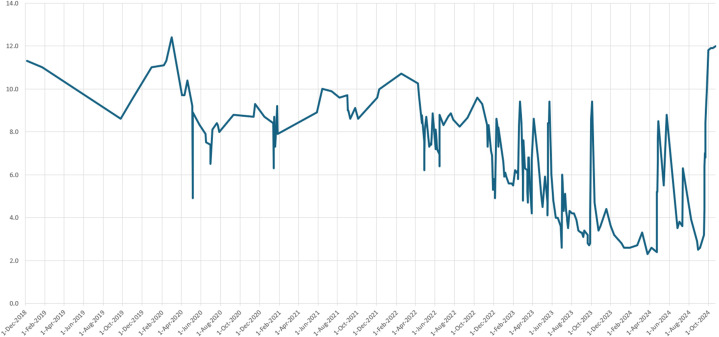
Haemoglobin Trend in g/dL.

Ms Y was noted to have a baseline haemoglobin level of 12.1g/dL in 2017. Since 2019, there were recurrent inexplicable episodes of severe iron deficiency anaemia, with the lowest haemoglobin level of 2.3 g/dL recorded in March 2024 (Refer **Figure 2**).

Haematology consults revealed an iron-deficiency anaemia of indeterminate origin following repeated extensive investigations across various time points in May 2023, September 2023, and May 2024 (refer **Table 1**). The case was extensively discussed at the Haematology grand rounds at multiple timepoints, arriving at the department consensus that no organic cause could be found.

**Table 1:** Diagnostic assessments and tests.

Investigation	Result (Reference range)
Serum iron	1.0 (9.0 - 30.4 umol/L)
Ferritin	<2 (5–204 ug/L)
Serum B12	235 (138–652 pmol/L)
LDH	221 (120–250 U/L)
Haptoglobin	0.85 (0.35 - 2.50 g/L)
Reticulocytes, absolute	49.9 (33.7-105.9 x 10E9/L)
Anti-tissue Transglutaminase Antibody IgA & IgG	Normal (<1.2 U/ml)
G6PD Qualitative	Normal
Parvovirus PCR	Negative
CT Abdomen & Pelvis	Right adnexal cyst. No haematoma or area of haemorrhage or source of bleeding identified
Oesophago-Gastro-Duodenoscopy	No evidence of ulcer, no active bleeding.
CT Enterography	Unremarkable
Meckel scan	Negative
Bone Marrow Aspiration	No evidence of leukaemia or lymphoma, absent iron stores is the main contributing factor for severe anaemia

Serial physical examinations revealed no new self-harm or venepuncture marks suggestive of bloodletting. A gynaecology consult and examination excluded gynaecological source of blood loss. Psychosocial investigations through family session provided limited information given the constraint relationship between Ms Y and her family members. Ms Y’s father was noted to be suffering from early aphasic dementia while Ms Y’s mother had limited interactions with her due to her own work schedule. Her mother shared that Ms Y was a picky eater since young and had largely avoided iron-rich food. The mother did not notice the presence of needles, syringes or apparatus at home that could potentially be used in bloodletting. Neither were there missing blood-thinners or medications that could be used to induce blood loss. A diagnosis of factitious anaemia, while likely, has proved to be difficult due to the lack of proof of obvious deception.

Multiple peer reviews, inter-disciplinary as well as inter-hospital meetings were conducted to clarify the diagnosis and subsequent management. This proved to be extremely challenging as Ms Y subsequently refused to provide consent to contact her family. She also did not consent to voluntary admissions either for psychiatric observation, or further medical treatments such as life-saving transfusions. Her mental capacity was frequently called into question, with psychiatrist specialists deeming her as mentally capacitated to make her own decisions.

### Management

Initial attempts to enforce treatment under the MCA proved to be onerous as her mental capacity was rapidly regained after the administration of life-saving blood transfusion. Ms Y also constantly contested to blood transfusions, citing reasonable concerns such as pain and fatigue from multiple venepuncture attempts as reasons for rejecting care. At one point, the haematologist acceded to Ms Y’s request for a Peripherally Inserted Central Catheter (PICC) in an attempt to increase compliance, but it was complicated by multiple line infections, with major concerns that she might be factitiously infecting the PICC line in order to increase contact time with the haematology receptionists and nurses with her pus-producing PICC. Our hospital legal team had also advised that as her haemoglobin levels increased with enforced blood transfusions, it would be difficult to justify using the MCA principles to enforce admission in our hospital against her will solely for the purpose of mental state observation and diagnosis of FD.

The treating psychiatrist then consulted the Adult Protection Officer (APO) in accordance with the VAA to discuss if the VAA could protect Ms Y from possible self-neglect. However, the APO found no grounds to subject her under the VAA as the mental infirmity based on her diagnosis of FD and Borderline Intellectual Functioning was not sufficient to enact the VAA for her.

As such, the treatment team consulted additional stakeholders, including the hospital legal team, as well as the IMH’s psychiatrists, to consider an involuntary admission under MHCTA as last resort due to the recurrent presentation of life-threatening anaemia. With much collaboration between all stakeholders, Ms Y was eventually re-admitted to our hospital to enforce involuntary admission under MHCTA to monitor her haemoglobin trend. The admission lasted 29 days and proved to be diagnostic as her haemoglobin rose without excessive medical intervention except for alternate day dosing of oral iron supplementation. Formal profiling of her intellectual functioning reported the following:

- Caution is advised in the interpretation of results as the assessment was conducted in English, which is a language that Ms Y is less proficient in. Her full-scale IQ (FSIQ) was not interpretable given the presence of significant index scatter (≥23 points difference between Verbal Comprehension Index (VCI) and Working Memory Index(WMI)).
- VCI assessed to be in Extremely Low range, Perceptual Reasoning Index and Processing Speed Index in Low Average range, WMI in average range
- Adaptive Behaviour Assessment System Third Edition (ABAS-3): Below Average range on the practical composite. As the informant, Ms Y’s mother did not observe some of the behaviour owing to constraint relationship, the remaining domains were not assessed in full.

Motivational interviewing was employed as main therapeutic focus while inpatient, highlighting the ambivalence between her desire to be independent and contribute financially through working versus her actual behaviours at that time.

Her haemoglobin level has remained stable post discharge and up till the time of manuscript submission. These observations have substantiated the diagnosis of FD. She continued to attend outpatient schema therapy after discharge.

### Patient’s perspective

Ms Y’s attitude towards treatment had been ambivalent throughout. Her reasoning was circular in nature, with no clear pattern or consistency. On one hand, she verbalised that she did not want to receive treatment as they were time consuming, costly, or painful. However, she was unemployed and her treatment was fully covered by financial assistance. On the other hand, she would comply when she was extremely symptomatic, and did not reject care when the MHCTA was enacted. She was neither agitated nor violent even during periods of uncooperativeness. Her mood was euthymic throughout, and she engaged the treating team in a sheepish and coy manner.

## Discussion

The diagnosis of FD was difficult to ascertain in this case owing to the chronicity and complex interplay of multiple psychiatric conditions elaborated above. Of note, there is significant overlapping clinical presentations between Borderline Intellectual Functioning (BIF) and FD. Patients with BIF may typically present with simplistic understanding of how their behaviours are risky, while patients with FD may superficially claim that they do not understand but display considerable ability for higher-order thinking in other areas. Careful observation of their interaction with other healthcare providers may shed some light into helping to differentiate the two diagnoses apart. However, our case highlights the ultimate challenge in treating patients with both conditions, which makes the clinical presentation much more complex and challenging.

This case was ethically challenging to manage as it sat at the intersection of the four key principles in medical ethics, namely beneficence, non-maleficence, autonomy, and justice (19, 20).

Autonomy vs Deception. Respect for the patient’s autonomy were complicated by the possibility of deception. Mental capacity evaluation, the crucial key to enforcing the MHCTA in this situation, was further complicated by her fluctuating medical status, Borderline Intellectual Functioning, longstanding psychiatric illnesses, and the lack of corroborative information from the family.

Beneficence and Non-Maleficence. The provision of appropriate care to Ms Y also had to be delicately balanced without enabling the maladaptive care-seeking and rejecting behaviours associated with FD. It was also challenging to balance the need for extensive workup to look for rare, undiagnosed diseases versus the possibility of unnecessary procedures arising from the deception of healthcare staff. Furthermore, the enforcement of lifesaving treatment could also be perceived as paternalistic and harmful for a childhood trauma survivor.

Justice. Some might argue that the use of MHCTA on FD might be controversial, as there is an absence of sufficient robust evidence to inform its definitive treatment, unlike other diagnoses such as Schizophrenia or Bipolar Disorder. However. at a broader level, not firming up Ms. Y’s diagnosis would mean that her excessive healthcare utilisation would continue to impact the fair distribution of limited healthcare resources to other patients in a resource-scarce country like Singapore.

### Legislation use in the Singapore context

This case sets the precedence for the exceptional application of the MHCTA outside of gazetted institutions in Singapore. The challenges included the lack of local or overseas precedence, and a clear framework in ethical decision weighing. The strengths in the clinical approach lies in the careful and extensive deliberation process involving the medical, psychiatric, and legal teams at both the institutional and national levels. This addressed the concerns of the continuity of both medical and psychiatric care.

This case has proven that the MHCTA is a useful statute to consider for future patients with similar FD and presentation. It may also be a catalyst in driving discussions between lawmakers, mental health professionals and protection officers from Ministry of Social and Family Development in pushing for amendments in other statutes such as VAA and MCA in safeguarding individuals with FD.

### Conclusion

Ms Y’s case had illustrated the practical difficulties of diagnosing FD and the utilisation of MHCTA as a last resort to solidify diagnosis of FD, on a balance of probability.

Involuntary admission maybe considered on a case-by-case basis in extenuating circumstances of diagnosing and managing severe FD given that it is potentially lifesaving. Further research addressing the mental capacity assessment of FD in refusing psychiatric treatment, the related ethical concerns, and the lasting gains of paternalistic treatment in FD would be helpful to shed light on the management of severe FD.

## Data Availability

The original contributions presented in the study are included in the article/supplementary material. Further inquiries can be directed to the corresponding author.
